# Leading the fight against liver cancer in Sub-Saharan Africa: developing the first multidisciplinary liver tumour board in Tanzania

**DOI:** 10.3332/ecancer.2025.1987

**Published:** 2025-09-15

**Authors:** Ally H Mwanga, Erick M Mbuguje, Jeanine Justiniano, Balowa Musa, Nashivai Kivuyo, Daniel W Kitua, Eva Uiso, Andrew Swallow, Edith Kimambo, Azza Naif, Deogratius B Mwanakulya, Swaleh Pazi, Advera Ngaiza, Seif Wibonela, Behnam Shaygi, Cameron E Gaskill

**Affiliations:** 1 Department of Surgery, Muhimbili University of Health and Allied Sciences, PO Box 65001, Dar es Salaam, Tanzania; 2 Muhimbili National Hospital, PO Box 65000, Dar es Salaam, Tanzania; 3 Department of Interventional Radiology, Muhimbili National Hospital, PO Box 65000 Dar es Salaam, Tanzania; 4 Department of Surgery, University of California Medical Center, 2335 Stockton Blvd, Sacramento, CA 95817, USA; 5 UC Davis Center for Global Surgery, 2335 Stockton Blvd, 6th Floor, Sacramento, CA 95817, USA; 6 Division of Gastroenterology, Department of Internal Medicine, Muhimbili National Hospital, PO Box 65000, Dar es Salaam, Tanzania; 7 Division of Oncology, Department of Internal Medicine, Muhimbili National Hospital, PO Box 65000, Dar es Salaam, Tanzania; 8 Division of Pathology, Department of Internal Medicine, Muhimbili National Hospital, PO Box 65000, Dar es Salaam, Tanzania; 9 Surgical Skills Laboratory, Muhimbili University of Health and Allied Sciences, PO Box 65001, Dar es Salaam, Tanzania; 10 London Northwest University Healthcare National Health Service Trust, Northwick Park Hospital, A404 Watford Rd, HA1 3UJ Harrow, UK

**Keywords:** liver tumour, delivery of health care, Tanzania, Sub-Saharan Africa, multidisciplinary team meeting

## Abstract

**Background:**

Multidisciplinary tumour boards (MTBs) are regular meetings of various specialist physicians and healthcare professionals who reach a consensus on diagnostic or therapeutic next steps in a cancer patient's care. They have been used for cancer care worldwide. The benefits of these MTBs are well established in the treatment of cancer, leading to higher rates of care completion and improved overall survival. The authors of this study set out to launch an MTB specifically for liver cancer in Tanzania.

**Methodology:**

In 2023, a multidisciplinary liver tumour board (MLTB) was established at Muhimbili National Hospital in Tanzania. This was designed to promote maximum engagement among medical specialties and international collaborators.

**Results:**

MLTB meetings were held weekly in-person and virtually, where members submit patient cases for review of clinical presentation, imaging, pathology and presumed diagnosis to allow discussion of next steps in diagnosis or treatment. A consensus recommendation is then communicated to the involved departments and the presenting physician proceeds with scheduling the patient for completion of the recommendations.

**Conclusion:**

This MLTB model aims to facilitate a comprehensive multidisciplinary treatment strategy for patients diagnosed with liver cancer. MLTBs are expected to enhance the quality of care provided to patients and promote the utilisation of advanced therapeutic options available in these nations.

## Background

### Introduction of multidisciplinary tumour boards (MTBs)

MTBs are regular meetings of specialist physicians and healthcare professionals involved in cancer care. During these meetings, patient cases are discussed to reach a consensus on diagnostic or therapeutic next steps in the patient's cancer journey [1]. MTBs have been shown to correlate with better stage classification, disease assessments, diagnosis, initial management plans and recommendations, higher rates of treatment, improved care processes, higher adherence to guidelines, shorter time to treatment and improved cancer outcomes [2]. The use of MTBs has become standard of care, and is often required, in the treatment of cancer in high-income countries, the effect of which could be an influencing factor in their better survival rates as compared to those in LMICs. Despite the immense potential of MTBs to influence cancer outcomes, their realized success and clinical impact are dependent on effective leadership and institutional support. If done correctly, MTBs improve coordination and communication among many disciplines and save time in patient care while fostering a learning environment for all involved. In Tanzania, attention has been focused on improving the care for liver cancer patients. The authors of this study set out to launch an MTB specifically for liver cancer in Tanzania.

### Liver cancer in Tanzania

Cancer is the 5th leading cause of death among adults in Tanzania, with an estimated 40,464 new cancer cases and 26,945 cancer-related deaths each year [3]. Among these, hepatocellular carcinoma (HCC) accounts for the 6th leading cause of cancer-related mortality. HCC is the predominant type of liver cancer in Africa, primarily driven by factors such as viral Hepatitis, aflatoxin exposure and alcohol consumption [4–7]. Jaka *et al* [8] described 142 patients with HCC in Mwanza, Tanzania, 88% of whom had delayed presentation in the Bugando Medical Center, none of whom received any treatment beyond supportive care, with 48% dying during their index admission. A regional cancer registry in the same region reported 140 patients with liver cancer, only 7 (5%) of which received care beyond palliative therapy [9]. At Muhimbili National Hospital (MNH) in Dar es Salaam, Tanzania, Mwanga *et al* [10] reported a cohort of 36 inpatients with HCC presenting with late-stage disease (83%) and advanced cirrhosis (41%), only 2 patients undergoing surgical resection. These studies highlighted the need for improved referral systems and access to liver cancer treatment throughout the country.

### Launching a multidisciplinary liver tumour board (MLTB) in Tanzania

The Tanzania Liver Cancer Group (TLCG) was created in 2022 as the first step in improving liver cancer care in Tanzania. The TLCG is a multi-institutional and multidisciplinary team with the mission to extend local and international awareness and promote collaborative research and care for liver cancer in Tanzania. This effort led to the inaugural Tanzania Liver Cancer Conference (TLCC) in March 2023 [11]. The TLCC was attended by 161 healthcare professionals from Tanzania and abroad, including 30 speakers from Tanzania, Kenya, Egypt, India and the United States. The conference focused on building awareness among local healthcare leaders and discussing the status of liver cancer care in Tanzania. The creation of the TLCG and the inauguration of the TLCC led to the initiation of open dialogue and conversations among healthcare professionals at MNH on how to create an MTB centred on liver cancer, to maintain momentum and solidify stakeholder engagement.

## Methodology

### Collaborations

Building on a collaboration around improving cancer care in Tanzania, MNH, Muhimbili University of Health and Allied Science (MUHAS), Road2IR foundation, the University of California, Davis (UC Davis), London North West University Healthcare NHS Trust, seed grants from Global Challenges Research Fund (GCRF) Networking Grants and European Union for Biomedical Imaging Research set out to establish the MLTB. Along with the local members within Tanzania, the MLTB also consists of members from international collaboration sites, including the United States of America, the United Kingdom, Malawi and Egypt.

### Institutions

The MLTB is based at MNH in Dar es Salaam, Tanzania. MNH is a national referral hospital and research centre, affiliated with the MUHAS. MNH has a 1,500-bed capacity and provides care to 2,000 outpatients per day, offering comprehensive healthcare services. Ocean Road Cancer Institute (ORCI) is a facility closely affiliated with MNH and is very involved in the MLTB meetings as well. ORCI provides care to 50,000 patients annually, 28,000 of whom are active cancer patients. They provide services such as prevention, early detection, diagnostics and therapeutics.

### Specialties in attendance

The first MLTB meeting took place in January 2023. The MLTB is composed of physicians from specialties, including hepatobiliary surgery, surgical gastroenterologists, general surgery, medical and clinical oncology, gastroenterology, hepatology, pathology, diagnostic radiology and interventional radiology. Meetings are also attended by fellows, residents and medical students from each specialty. Along with the above physicians, allied healthcare professionals available at the meetings include physiotherapists, nutritionists, information technology and Cancer Registry Data Team whenever possible.

### Funding

The MLTB primarily obtained essential funding through international grants, specifically from the European Society of Radiology and the GCRF. These grants were instrumental in addressing key operational needs, including hiring a dedicated Research Assistant to manage documentation and coordination of MLTB activities, and acquiring necessary equipment like computers and an advanced audio-conferencing system. In addition, the TLCG facilitated the acquisition of a large wall-mounted video screen, greatly improving the quality of real-time case presentations and interactive discussions during meetings.

Despite these successes, securing funds posed significant challenges. Local funding was scarce, which increased dependence on international sources. International grants had complex and lengthy application procedures, often resulting in delays in project start dates and equipment procurement. Additionally, high costs associated with advanced technological resources meant that purchases had to be carefully prioritized based on immediate needs.

MNH significantly alleviated some financial pressures by providing crucial support such as meeting spaces and fundamental infrastructure. The presence of a Research Assistant with IT expertise further streamlined the setup and maintenance of sophisticated conferencing and IT systems, greatly enhancing the MLTBs’ operational efficiency and sustainability.

### Resources

#### Physical space

The MLTB meetings are held in a conference room at MNH weekly. The room has ample capacity for more than 15 specialists who attend the meetings in person. It has air conditioning, good lighting and large windows with curtains that can be drawn to reduce light when reviewing images.

#### Technology and equipment

The conference room is equipped with a computer desktop and a large wall-mounted screen, as well as several speakers and microphones. The screen is connected to the computer desktop, which allows for ease of presentation. The computer itself is connected to the hospital network and equipped with the software necessary to project PowerPoints and radiographic images. Wireless and hardwired internet connections allow the computers to connect to the virtual meeting platform. Additionally, research coordinators can collect data on patient health information, presentation details and tumour board metrics that are stored on the tumour board database via the Liver Cancer Registry RedCap.

## Results

### MLTB meeting preparations

Physicians from any specialty may submit cases for review of any patient with a presumed diagnosis of HCC or other liver pathology. Case submission is coordinated through the MLTB administrator and research assistant. Physicians communicate with the MLTB administrator to initiate the process, either through direct lines or a coordinated WhatsApp group of MLTB participants. The coordinator provides the physician with a PowerPoint presentation template, which ensures that standardized essential information is included in the presentation, such as the patient’s clinical history; laboratory results, including virology, hepatic function panel, tumour markers and coagulation factors; pathology results; as well as radiology imaging and reads. This template aligns with the official Liver Cancer Registry and ensures that the presented data are consistent and complete. Additionally, the necessary imaging for the patient must be available, either on CD or through direct access to the hospital’s imaging system. The MLTB administrator and a member of the IR team make sure that the images are ready for review prior to the MLTB meeting. The presentation for each case is shared with the MLTB members in advance of the meeting to allow individual comprehensive review of the case prior to multidisciplinary discussion. This ensures that all team members are familiar with the clinical history and key details, enabling a more informed discussion during the meeting.

### During the MLTB

The meeting begins with general remarks and announcements by the MLTB leadership. An attendance sheet is distributed, allowing tracking of requested continuous professional development points and tracking the active members of the tumour board. The MLTB leadership will officiate the meeting, confirming representation of each specialty is in attendance and leading the group through the scheduled agenda. Each case presentation consists of the 5-minute PowerPoint presentation from a resident/physician presenting a patient, reviewing clinical presentation, laboratory results, radiologic reads and presumed diagnosis, with a question to the board on how to proceed with evaluation or treatment. The imaging is projected, and the diagnostic and interventional radiology team reviews the imaging in real time to the rest of the MLTB. Once all members are satisfied with the review, the MLTB discusses the case and arrives at a final decision regarding the patient’s diagnosis and treatment plan, with recommendations on further diagnostics or therapies. The MLTB leadership will facilitate discussion of each case as needed, calling for remarks from each specialty prior to determining the consensus recommendation. The meeting usually consists of 1–5 patient presentations and closes with updates on patients previously discussed.

On occasion, there are educational presentations on select topics pertaining to liver cancer, often assigned to resident physicians at MNH or by international collaborators dedicated to global surgery research to aid overall conference education. In addition, there is space for physicians to present any upcoming or current research project proposals and updates related to liver cancer and the patients presented at the MLTB. This allows expansion opportunities in academic medicine related to liver cancer in Tanzania.

### After the MLTB

The final consensus decision is recorded in the Liver Cancer Registry. The consensus plan is communicated to the involved departments, and the presenting physician proceeds with scheduling the patient for any additional diagnostic or therapeutic treatments. Patient updates are communicated to the MLTB during subsequent meetings or the MLTB WhatsApp group and transcribed in the patient’s Liver Cancer Registry information with the pertinent follow-up information. We currently have a Liver Cancer Registry Database on the MNH RedCap that serves as a centralized case database on all liver cancer patients who have presented to MNH. However, it does not include liver cancer patients from other institutions in Tanzania yet.

Since the beginning of the tumour board in January 2023, there have been 265 patient cases presented. Our success metrics are measured by the engagement and attendance at the weekly tumour board meetings per specialty; 100% of our tumour board meetings have been attended by a surgeon, interventional radiologist and tumour board administrative personnel. Gastroenterology and oncology specialists are present 90% and 85% of the time, respectively. The presence of pathologists was implemented at the end of 2024; consequently, their attendance was low at 15% of tumour boards since January 2023. Each meeting is attended by at least 2 subspecialties; therefore, 100% of the presented patients in the tumour board receive multidisciplinary input. Currently, there is no integration of the tumour board within the existing hospital systems (MNH and ORCI). There have been conversations with the medical director at MNH to have the tumour board consensus diagnosis and recommendation be input into the electronic medical record, but this has not been adopted yet.

## Discussion

### Benefits and impact

The MLTB at MNH greatly impacted liver cancer care in Dar es Salaam. Having regular meetings with multiple specialties involved to reach consensus recommendations led to improved care processes, higher adherence to international oncologic guidelines and faster treatment referrals.

The learning environment at the meetings has encouraged learning among all the specialties and at all levels of training, as well as inspired research endeavours to improve the healthcare being offered to patients in Tanzania. Many international collaborative relationships arose from the MLTB with a joint mission to improve access to cancer care.

### Lessons learned and hardships overcome

Despite the recognized importance, dedicated leadership and institutional support of the MLTB, implementation has not proceeded without overcoming barriers. Stakeholder engagement of the required specialties is the key to tumour board success, supporting regular attendance, participation and meaningful discussion. While MUHAS houses most of the participating departments, medical oncology is centred several miles away at ORCI, making in-person attendance difficult. This has been overcome by the utilisation of virtual meetings, with the added benefit of allowing others who are off-campus or international to participate. Reliance on the virtual platform, however, comes with its own difficulties, as lapses in technology and wavering internet connectivity can hinder MLTB discussions and cause unnecessary delays in the agenda. We have dedicated significant resources to improving the connectivity to our MLTB conference room to successfully overcome these issues. Additionally, despite the benefits of the virtual platform allowing outside participants, the technology needed to transfer high-resolution radiology images and digital pathology slides is not routinely available. This severely hinders the thoroughness of discussion for remote cases, limiting expansion of the tumour board beyond the hosting institutions. Utilizing the MLTB coordinator to collect and prepare material prior to each meeting has helped to overcome these issues; however, a more streamlined process will need to be established. For example, imaging sharing has been limited because of technological difficulties with the imaging server and database used at MNH. We have been able to solve this by having the presenting physician bring a copy of the imaging on a CD-ROM ahead of time and make sure that it works in the conference room desktop prior to the meeting.

Currently, participation in the MLTB is largely limited to MUHAS and ORCI personnel, presenting patients from MNH and ORCI. Access to treatment and resources are limited in the periphery, and care recommendations vary by location, resulting in delayed, disjointed and uncoordinated care [11]. Early information on treatment describes only 1%–5% of HCC patients in Tanzania receiving any curative treatment [8, 10]. Surgical resections account for all of these, as ablation and transplants are unavailable in Tanzania currently and access to immunotherapy is limited. While trans-arterial therapy capacity is present, delivery and outcomes metrics are unpublished. Most patients receive supportive measures only. Therefore, final recommendations are limited to treatments available in these facilities, without the capacity to offer or coordinate care for patients who live outside of the area. Regardless of the regional scope of the MLTB, there is widespread national interest in expanding the MLTB to other facilities.

Communication between facilities and between physicians has also been a challenge. Once a consensus has been determined by the MLTB after a patient is presented, it is unclear how that decision is communicated to the patient’s other physicians. We are currently standardizing documentation of the MLTB discussion within the electronic medical record. By incorporating MLTB recommendations using official documentation templates, MLTB consensus recommendations could be accessed by all physicians involved in a patient’s care, improving both care coordination and communication among providers. Follow up on whether the treatment is carried out in a timely fashion is completely dependent on the involved physicians and loss to follow up is a significant risk. There is also limited ability to follow patient outcomes resulting from the consensus recommendation. A solution to this issue is currently still pending.

Finally, patient involvement in the MLTB decision-making is still a work in progress. There is a need to involve the patients more formally when the MLTB is discussing their case and whether the patient agrees with undergoing the treatment recommendation or if there are any barriers limiting the patient from having access to the recommended treatment. An effort to address this is underway, with the planning of a post-MLTB clinic to convey and discuss the consensus recommendations with the patient or the next of kin in a formalized manner.

### Future directions

Establishing an MLTB for liver cancer care in Dar es Salaam, Tanzania, has led to many different projects. The expansion of the current MLTB is a current priority, as we are constantly recruiting additional physicians from all specialties to be involved in the MLTB at MNH. We would also like to improve the long-term outcome tracking of past and future liver cancer patients who have been presented at the MLTB, to better assess the impact the MLTB has made thus far.

Expanding this model to cover the entire country will facilitate screening programs, early detection of HCC patients and treatment options at appropriate times, including liver resection, local regional therapy, systemic therapy and eventually possible liver transplant. National tumour boards have demonstrated feasibility in several LMICs, including within East Africa, utilizing telepathology and video conferences to increase participation access [11–13]. The MLTB at MNH/MUHAS will set out to implement a national MLTB that will be informed by a nationwide assessment of HCC diagnostic and treatment capacity, designed to also identify key national stakeholders for inclusion in the board. Funding for this project will be coming from a grant provided by the University of California. We are currently in the process of receiving ethical approval from different institutions and hope to begin these efforts, which will lead to an in-depth capacity assessment of the Tanzania cancer care health systems during our TLCC 2025 meeting.

### Conclusion

The prevailing model of the MLTB, which is currently implemented at MNH and MUHAS, possesses the potential for nationwide application as well as implementation in any LMIC. This model aims to facilitate a comprehensive multidisciplinary treatment strategy for patients diagnosed with HCC. Consequently, it is expected to enhance the quality of care provided to patients and promote the utilisation of advanced therapeutic options available in these nations, including liver resection, local regional interventional radiology therapies, liver transplantation and immunotherapies.

## Conflicts of interest

No conflicts of interest.

## Author contributions

Conceptualisation: (initials) AHM, EMM, CG, JJ and SW.

Methodology: (initials) AHM, EMM, CG, JJ, BM, NK, DWK, EU, AS, EK, AN, SP, SW and BS.

Writing–original draft: (initials) AHM, EMM, CG, JJ and SW.

Writing–review and editing: (initials) AHM, EMM, CG, JJ, BM, NK, DWK, EU, AS, EK, AN, SP, SW and BS.

All authors have read and approved the final manuscript.
